# aMMP-8 Point-of-Care/Chairside Oral Fluid Technology as a Rapid, Non-Invasive Tool for Periodontitis and Peri-Implantitis Screening in a Medical Care Setting

**DOI:** 10.3390/diagnostics10080562

**Published:** 2020-08-05

**Authors:** Hanna Lähteenmäki, Kehinde A. Umeizudike, Anna Maria Heikkinen, Ismo T. Räisänen, Nilminie Rathnayake, Gunnar Johannsen, Taina Tervahartiala, Solomon O. Nwhator, Timo Sorsa

**Affiliations:** 1 Department of Oral and Maxillofacial Diseases, Head and Neck Center, University of Helsinki and Helsinki University Hospital, PO Box 63 (Haartmaninkatu 8), FI-00014 Helsinki, Finland; hanna.maa.lahteenmaki@gmail.com (H.L.); anna.m.heikkinen@helsinki.fi (A.M.H.); rathnayake.nilminie@helsinki.fi (N.R.); taina.tervahartiala@helsinki.fi (T.T.); timo.sorsa@helsinki.fi (T.S.); 2 Department of Preventive Dentistry, Faculty of Dental Sciences, College of Medicine, University of Lagos, Lagos 100213, Nigeria; kumeiz09@gmail.com; 3 Division of Periodontology, Department of Dental Medicine, Karolinska Institutet, SE-171 77 Stockholm, Sweden; gunnar.johannsen@ki.se; 4 Department of Preventive & Community Dentistry, Faculty of Dentistry, College of Health Sciences, Obafemi Awolowo University, Ile-Ife A234, Nigeria; periodontologist2010@gmail.com

**Keywords:** periodontitis, peri-implantitis, screening, point-of-care testing, biomarkers, aMMP-8, biosensors, point-of-care diagnostics

## Abstract

This communication article addresses currently available rapid non-invasive methods to screen and detect periodontitis and dental peri-implantitis. In this regard, oral fluid biomarkers have been researched extensively but self-reported oral health (SROH)-questionnaires have also been developed. Both alternatives may offer a quick and easy way to screen and detect diseased patients. Active matrix metalloproteinase (aMMP-8) is one of the most validated biomarkers for screening and detecting periodontal breakdown related to periodontitis and peri-implantitis and monitoring their treatment effects revealing successful, less- and non-successful treatment results. Currently available aMMP-8 lateral-flow technologies allow this kind of analysis, as demonstrated here, to be conducted quantitatively online and real-time as point-of-care/chairside testing in dental and even medical care settings. In this study, an aMMP-8 peri-implant sulcular fluid point-of-care-test diagnosed peri-implantitis and healthy implants far more accurately than bleeding-on-probing or the other biomarkers, such as polymorphonuclear (PMN)/neutrophil elastase, myeloperoxidase and MMP-9. Although, SROH-questionnaires allow screening in similar settings but they lack the information about the current disease activity of periodontitis and peri-implantitis, which is of essential value in periodontal diagnostics and treatment monitoring. Thus, both methods can be considered as adjunct methods for periodontitis and peri-implant diagnostics, but the value of oral fluid biomarkers analysis does not seem to be substitutable.

## 1. Introduction

Salivary/oral fluid biomarkers such as active matrix metalloproteinase-8 (aMMP-8) are becoming increasingly useful in the rapid diagnosis of periodontitis and dental peri-implantitis as documented in several studies [1,2,3]. Proteolytic enzymes capable of degrading connective tissue, as well as their activators and inhibitors, have been a natural target to identify promising candidates causing loss of attachment in both periodontitis and peri-implantitis. These include but are not limited to matrix metalloproteinase-8 (MMP-8, neutrophil collagenase), matrix metalloproteinase-9 (MMP-9, gelatinase B), polymorphonuclear leukocyte (PMN)/neutrophil elastase, myeloperoxidase (MPO) and tissue inhibitor of metalloproteinases 1 (TIMP-1). Recently, the world workshop on periodontal and peri-implant diseases classifications emphasized the utilization of validated biomarkers in the case definition systems [4,5]. Recently, Sorsa et al. [6] especially implicated the oral mouthrinse aMMP-8 as the needed biomarker for the latest periodontitis classification system [4]. Such oral fluid biomarkers are now commercially available as quantitative point-of-care (PoC) kits for periodontitis and dental peri-implantitis [1,2,3]. Examples regarding aMMP-8 are the quantitative reader equipped oral fluid (mouthrinse, gingival crevicular fluid (GCF), and peri-implant sulcular fluid (PISF)) aMMP-8 lateral-flow chairside/PoC tests (PerioSafe and ImplantSafe with ORALyzer kits) [7], which have been successfully demonstrated in numerous studies globally [6,7,8,9,10,11,12]. The MMP-8/aMMP-8 is a promising candidate biomarker to diagnose, predict, grade periodontitis/peri-implantitis and monitor their treatments [6,7,8,9,10,11,12,13,14,15,16,17,18,19,20,21]. The aMMP-8 test kits have been independently validated in Europe, United States and Africa [6,7,8,9,10,11,12] and also shown to be useful in medical care settings such as in detecting prediabetes and diabetes at the dental office [22].

Recently, Verhulst et al. [23] demonstrated a self-reported oral health (SROH) questionnaire to be an easy and quick periodontitis screening method which was commendable and an alternative screening to biomarkers in oral rinse samples. They also reported total MMP-8, not aMMP-8, as the only salivary biomarker (out of four biomarkers evaluated) that was not associated with periodontitis. Although the authors were surprised by this outcome [23], this could have been due to the evaluation of total MMP-8 instead of aMMP-8 [23,24]. Perhaps, the assessment of the catalytically competent aMMP-8 instead of total MMP-8 would have shown a direct correlation with periodontal disease severity and treatment monitoring [6,7,8,11]. Verhulst et al. [23] also acknowledged that analyzing aMMP-8 rather than the protein concentration in saliva would have been an alternative choice, as has been reported by other authors [6,7,8,9,10,11,12,24]. The sensitivity and specificity of the SROH questionnaire for total periodontitis was 85% and 63% while for severe periodontitis, was 65% and 81% [23]. Evidence from some studies suggests that higher sensitivity (96%) for poor oral hygiene, 95% for chronic periodontitis and 82.6% for at least two sites with bleeding on probing (BOP) based on aMMP-8 oral fluid PoC tests can be achieved, hence may be superior to SROH questionnaires [8,23,25].

Currently, there are few peri-implantitis studies comparing aMMP-8 PoC technologies and biomarkers of peri-implantitis. Thus, one of the aims of this study is to assess the accuracy of aMMP-8 PISF POC test (ImplantSafe) in comparison to several candidate biomarkers of peri-implantitis. Another aim of this study is to evaluate the value of aMMP-8 lateral-flow PoC technologies in medical and dental settings for screening and detecting periodontally diseased patients and peri-implantitis patients in a non-invasive way. In this regard, an example of monitoring the treatment of periodontitis patients using aMMP-8 mouthrinse POC test (PerioSafe) is presented as well.

## 2. Materials and Methods

### 2.1. Patient Selection and Characterization

This clinical study was approved by the local ethical committee of Karolinska Institutet, Sweden (2016-08-24/2016/1:8 and 24 January 2016.), and the Helsinki University Central Hospital, Finland (nro260/13/03/00/13, 17 December 2013). The dental implant patient participants (*n* = 52) were recruited from the Danakliniken (specialist clinic), Stockholm and Karolinska Institutet, Sweden. The periodontitis patient participants (*n* = 15) were recruited from the Capital region and Kirkkonummi unit for special dental care, Helsinki, Finland. Participants provided a written informed consent in this study.

At baseline 26 patients with peri-implantitis and 26 randomly selected controls free from peri-implantitis and periodontitis were included (Table 1). People above 18 years old were included in this study. Peri-implantitis was diagnosed as probing pocket depth (PPD) ≥ 4 mm, BOP and bone loss ≥ 2 mm. Peri-implantitis was graded according to alveolar bone loss and peri-implant inflammation assessed as the number of pathological pockets (gingival pockets ≥4 mm) and mucosal inflammation [26]. Controls were selected if they were free from peri-implantitis and periodontitis. At baseline, the controls were investigated in the same way as the peri-implantitis patients. All participants in this investigation were asked to respond to a validated questionnaire, comprising of various questions related to family history, medication, previous diseases in particular inflammatory disorders, diet, smoking, pulmonary diseases, socioeconomic factors, stress and information about general oral health status.

A standardized dental examination, including X-rays, was performed and collection of PISF samples using PoC tests were performed. Participants were examined clinically for number of teeth and implants present, soft tissue pathologies and periodontal status, including PPD of six sites per implant/tooth, BOP and plaque score as present or absent, mobility and furcation involvement (teeth) were recorded. A standard millimeter graded Hu-Friedy probe (Hu-Friedy Manufacturing Co., LLC, Chicago, IL, USA) was used to record the PPD around the implants. The periodontal status of all participants was classified according to clinical and radiographic criteria, such as number of pathological pockets ≥4 mm and gingival inflammation. Digital intra-oral/bitewing/panoramic radiographs were taken at the regular check-ups of all participants by the dentist.

Fifteen periodontitis patients (8 males and 7 females) aged 34 to 65 years were recruited and their periodontal status was classified according to clinical and radiographic criteria, such as number of pathological pockets ≥4 mm, gingival inflammation and alveolar bone loss (see Table 2). One of the patients had type II diabetes, one had Chron’s disease and epilepsy and one had depression; these patients had appropriate medication for these diseases. A standard WHO probe was used to record periodontal status. All the patients received anti-infective treatment/scaling and root planing (SRP) at baseline and at 4 weeks (1st recall visit). The effect of the periodontal treatment was monitored using an aMMP-8 mouthrinse PoC test kit (PerioSafe-ORALyzer kit by Medix Biochemica Ltd, Espoo, Finland) at baseline, 4 weeks (1st recall visit) and 8 weeks (2nd recall visit) according to manufacturer’s instructions as described earlier [7,8]. Patients received the periodontal treatment after the mouthrinse samples were collected and analyzed by the aMMP-8 test kit. Seven periodontitis patients decided to end the periodontal treatment monitoring program after 4 weeks (1st recall visit) for unknown reasons.

### 2.2. PISF Sample Collection

PISF sample collection was conducted step by step according to the manufacturer’s (ImplantSafe aMMP-8 PoC test by Medix Biochemica Ltd., Espoo, Finland) instructions. The sampling site was prepared by removal of excess saliva with a short, gentle blast of air. A sterile PISF collection strip was placed apically as deeply as possible into the sulcus at the sampling site using the tweezers. The strip was left there for 30 s and was then pulled off with a carrier and placed in the buffer vial for elusion. The vial was gently turned upside down 5 times. After waiting for 5 min to make sure the PISF collection strip was totally immersed in the fluid, the tube was once again pivoted 5 times by turning it carefully upside down.

### 2.3. Peri-Implant Crevicular Fluid Analysis (aMMP-8 PoC Test and Other Biomarkers)

aMMP-8 PoC ImplantSafe kit samples were analyzed by dipstick, which was dipped with the yellow absorption zone into the elution fluid until the liquid was visible in the readout window. This took 12–15 s. After that the dipstick was removed from the elution fluid and placed on a level surface. The sample first appeared as a blue haze in the readout window, after which the control line “C” and eventually the test line “T” appeared. The results were read after exactly 5 min. For the evaluation, the test strip was placed on a horizontal surface. Collected PISF samples were also analyzed for aMMP-8 by immunofluorometric assay (IFMA); human PMN elastase, MPO, TIMP-1 by enzyme-linked immunosorbent assay (ELISA) and pro- and active MMP-9 by gelatin-zymography as described previously [6,27].

### 2.4. Statistical Analysis

Basic clinical characteristics were summarized for study patients with dental peri-implantitis and their healthy controls. Comparisons between peri-implantitis and control groups were performed using nonparametric Mann–Whitney U test (the results were equivalent to *t*-test) and Fisher’s exact test. Receiver operating characteristic (ROC) analysis and the area under the ROC curve (AUC) were used for studying the diagnostic ability of the biomarker candidates to discriminate peri-implantitis from a healthy implant. The Youden index was used defining the best cut-offs from the ROC curves for each biomarker. Diagnostic sensitivity (Se), specificity (Sp), the percentage of false negatives (FN) and false positives (FP), test accuracy (Acc) and Matthews correlation coefficient (MCC) were calculated by using the best cut-off to assess the quality of classification of dental peri-implant health and disease. The effect of periodontal treatment on the 15 adult patients with periodontitis was compared by Friedman test in (the three time points) and by Wilcoxon signed ranks test (base level vs. 1st visit). A two-tailed *p*-value < 0.05 was set as statistically significant. Statistical analyses were performed using SPSS Statistics, version 25 (IBM Corp, Armonk, NY, USA).

## 3. Results

**Figure 1:**
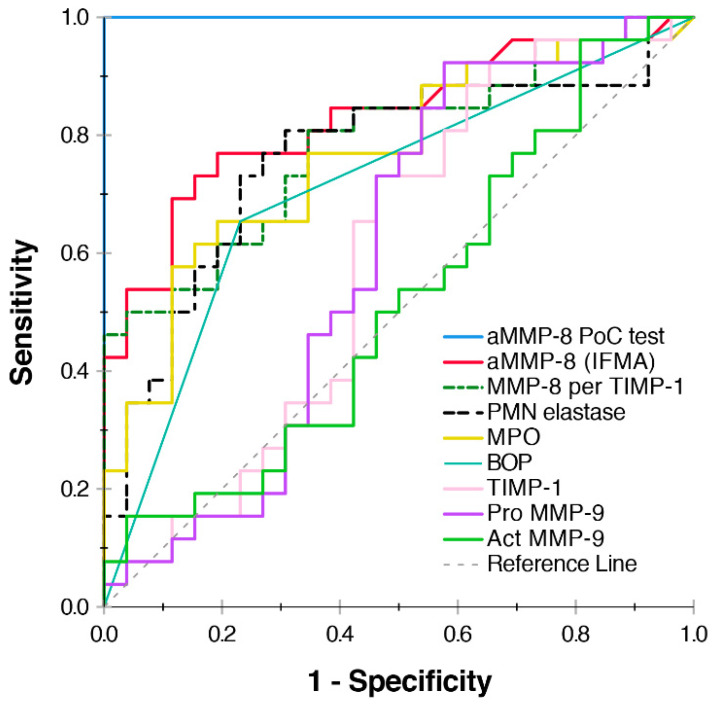
Receiver operating characteristic (ROC) analysis illustrating the diagnostic ability of the biomarker candidates to discriminate peri-implantitis from healthy implant (26 peri-implant and 26 healthy dental implant patients). aMMP-8 PoC test, IFMA, ELISA and gelatin-zymography for indicated biomarkers and bleeding on probing (BOP) were assessed as described previously [7,12,26,27].

The aMMP-8 PoC kit correctly diagnosed all 26 peri-implantitis and 26 healthy implant patients (Figure 1, Table 1 and Table 3). Thus, the aMMP-8 ImplantSafe-ORALyzer kit detected peri-implantitis-affected sites more efficiently than aMMP-8 (IFMA), MMP-8/TIMP-1, PMN elastase, MPO, TIMP-1, actMMP-9 and proMMP-9 and BOP as illustrated by the ROC analysis in Figure 1 and Table 3. Furthermore, it should be noted that, in addition to aMMP-8 PoC test, there were also several other biomarkers that were more efficient than BOP (Figure 1, Table 3).

**Figure 2:**
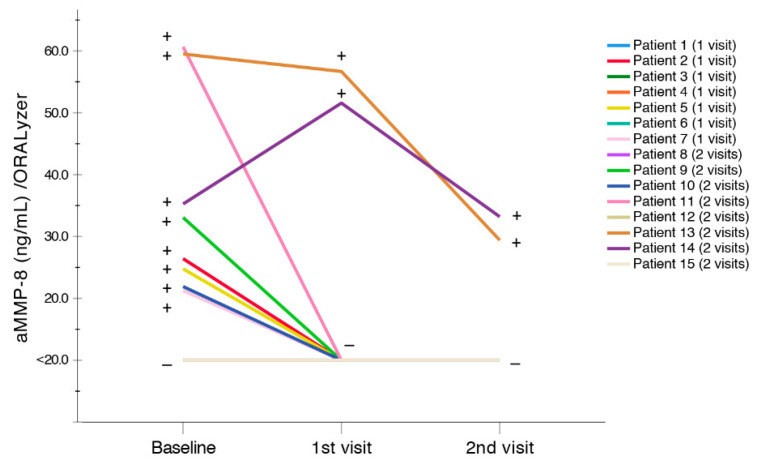
Effects of periodontal treatment; anti-infective treatment/scaling and root planing (SRP) in 15 adult periodontitis patients with elevated aMMP-8 (+, >20 ng/mL) in their mouthrinses monitored by aMMP-8 PoC-system (PerioSafe-ORALyzer kit) for 1st recall visit (4 weeks) and 2nd recall visit (8 weeks). (−) indicates <20 ng/mL aMMP-8 in mouthrinse.

The aMMP-8 PoC systems can also do an on-line and real-time quantitative monitoring of successful (−), less- and non-successful (+) periodontal treatment effects of anti-infective treatment/SRP (Figure 2). In Figure 2, SRP reduced most (11/15) of the elevated (+) aMMP-8 levels to (−) below 20 ng/mL (cut-off) while 2/15 of cases reduced but remained elevated (+) at the 1st and 2nd recall visits in the periodontitis patients [7]. As a whole, the effect of periodontal treatment was significantly positive on the clinical parameters among the majority of the periodontitis patients (Table 2).

## 4. Discussion

The main finding of this study was that the aMMP-8 PISF PoC test was far more accurate in diagnosing peri-implantitis and healthy implants than BOP or the other biomarkers of this study. Furthermore, aMMP-8 levels measured by IFMA had the second-best classification ability, but also MMP-8 per TIMP-1, PMN elastase and MPO showed better potential to classify peri-implant disease and health than BOP. On the other hand, act MMP-9, pro MMP-9 and TIMP-showed lower potential than BOP. Thus, in addition to aMMP-8, MMP-8 per TIMP-1, PMN elastase and MPO, seem to be possible alternatives for BOP as more accurate indicators of peri-implant tissue destruction and peri-implantitis. However, more research is still needed in this regard to confirm this.

Diagnosing periodontitis and peri-implantitis is mainly based on clinical and radiographic examination, which assess the extent and severity of periodontitis and peri-implantitis. However, their accuracy in monitoring disease activity, progression and treatment effects is still limited, and, in this regard, biomarkers, such as aMMP-8, could improve the accuracy of oral periodontal and peri-implant disease diagnostics [2,3,4]. On the other hand, SROH questionnaires could be beneficial screening tools for periodontitis and peri-implantitis, as well, especially related to the important risk factors, such as smoking and diabetes [4,5], but also in settings where oral rinse and PISF tests or professional dental equipment and expertise are unavailable. However, the scientifically proven value of using validated, rapid oral rinse and PISF PoC aMMP-8 biomarkers [6,7,8,9,10,11,12,13,14,15,16,17,18,19,20,21] to detect online and monitor quantitatively periodontitis and peri-implantitis and their disease activity, progression and treatment effects is something that should not be undermined when screening for these diseases in dental or medical settings. In this study, the aMMP-8 mouthrinse test measuring the collagenolytic activity indicated successful, less- and non-successful periodontal treatment effects of anti-infective treatment/SRP in patients with periodontitis (Figure 2). We additionally wish to note that the ease-of-use of the technology matters as well. For example, the method described by Verhulst et al. [23] for the oral fluid collection coupled with the laborious procedures, including the time-requiring overnight protocol, for the biomarkers utilized may not be so rapid compared to a quantitative aMMP-8 PoC system such as PerioSafe/ImplantSafe oral fluid tests which have only 5–6 min chairside turn around (Figure 1 and Figure 2, Table 3) [6,7,12]. Furthermore, in this study, aMMP-8 PISF PoC test had significantly better discrimination of peri-implantitis disease than MMP-9 and other biomarkers that, to our knowledge, do not currently have rapid point-of-care/chairside technologies available.

Although this study was conducted in dental care settings, the application of validated aMMP-8 PoC/chairside tests [6,7,8,9,10,11,12] could be of scientific value in point-of-care diagnostics of periodontitis and peri-implantitis in medical care settings as well. There is evidence of a link between severe periodontitis and several systemic diseases, such as diabetes and cardiovascular diseases [28,29,30]. For example, the inflamed periodontal tissues release pro-inflammatory cytokines and tissue destruction mediators into the circulation system increasing the systemic inflammatory burden [31]. Furthermore, periodontitis has a two-way relationship with diabetes [29]. Poor periodontal condition may increase, for example, the risk of insulin resistance and diabetes complications [29]. In this regard, a recent study demonstrated that oral rinse aMMP-8 PoC/chairside test can also be used for detecting and screening prediabetes and diabetes in addition to periodontitis in the dental office [22]. As such, aMMP-8 PoC/chairside tests could also be useful for medical professionals for assessing the risk of ongoing active periodontal breakdown and its possible systemic effects among their (diabetes) patients. These risk patients could be referred to a dentist for a periodontal examination and evaluation of their treatment need [28,29]. Finally, recent studies indicate that diabetes is a risk factor for severe COVID-19 infections [32]. Regarding the association between periodontitis and diabetes, biomarkers, such as aMMP-8, have been suggested to be utilized in the identification of patients with diabetes and active periodontal disease for referring them to targeted prevention for COVID-19 [33,34]. In conclusion, the relationship between periodontitis and several systemic diseases offers many possibilities for future studies regarding aMMP-8 oral fluid testing and other biomarkers linking dental and medical professionals.

## Figures and Tables

**Table 1:** Demographics of the study population.

Condition/Variable	Peri-Implantitis (*n* = 26)	Controls (*n* = 26)	*p*-Value
Age, mean ± SD	67.31 ± 10.97	59.38 ± 9.39	0.007
Gender, *n*			
Female	14	19	0.249
Male	12	7	
Smoking, *n*			
Yes	2	8	0.075
No	24	18	
PPD < 4 mm	0	20	<0.001
PPD = 4–5 mm	4	5	
PPD > 5 mm	22	1	
PPD (mm), mean ± SD	7.77 ± 1.92	3.35 ± 0.75	<0.001
Plaque, *n*			
Yes	10	0	0.001
No	16	26	
BOP, *n*			
Yes	17	6	0.005
No	9	20	
Calculus (subgingival), *n*			
Yes	0	2	0.49
No	26	24	
Suppuration, *n*			
Yes	15	0	<0.001
No	11	26	
CVD, *n*			
Yes	6	3	0.465
No	20	23	
Hypertension, *n*			
Yes	4	8	0.324
No	22	18	
Diabetes, *n*			
Yes	2	3	1
No	24	23	
Osteoporosis, *n*			
Yes	1	0	1
No	25	26	
Renal diseases, *n*			
Yes	1	1	1
No	25	25	
Rheumatoid arthritis, *n*			
Yes	1	2	1
No	25	24	

PPD: probing pocket depth; BOP: bleeding on probing; CVD: cardiovascular disease; for clinical parameters plaque, BOP, calculus, suppuration yes/no: present/absent on the implant sites. *p*-value calculated continuous variables by Mann–Whitney test (exact, 2-sided) and for categorical variables by Fisher’s exact test (2-sided).

**Table 2:** Effects of periodontal treatment; anti-infective treatment/scaling and root planing (SRP) in 15 adult patients with periodontitis to the clinical parameters (the number of PPD ≥4 mm, CAL mean, BOP) examined base level (*n* = 15), 1st recall visit (4 weeks, *n* = 15) and 2nd recall visit (8 weeks, *n* = 8).

Periodontal Parameters	Baseline (*n* = 15)			1st Visit (*n* = 15)			2nd Visit (*n* = 8)				
	Min	Max	Mean (SD)	Min	Max	Mean (SD)	Min	Max	Mean (SD)	*p*-Value *	*p*-Value **
The Number of PPD ≥ 4 mm	29	132	73.8 (35.3)	10	114	36.1 (30.6)	10	65	25.3 (18.1)	0.001	0.001
CAL (mm) Mean	3.6	5.6	4.5 (0.6)	2.8	5.2	4.0 (0.6)	4.3	5.9	5.1 (0.6)	0.072	0.004
BOP (%)	13	75	43.9 (18.0)	9	38	19.8 (7.7)	5	44	20.1 (12.0)	0.001	0.001

PPD: probing pocket depth; CAL: clinical attachment loss; BOP: bleeding on probing (full mouth). * Friedman test, asymptotic, 2-sided (baseline, 1st visit and 2nd visit, *n* = 8); ** Wilcoxon signed ranks test, asymptotic, 2-sided (base level and 1st visit, *n* = 15).

**Table 3:** Receiver operating characteristic (ROC) analysis and the area under the ROC curve (AUC) illustrating the diagnostic ability of the biomarker candidates to discriminate peri-implantitis from a healthy implant (26 peri-implant and 26 healthy dental implant patients). The Youden index was used defining the best cut-offs from the ROC curves of biomarkers. Diagnostic sensitivity (Se), specificity (Sp), the percentage of false negatives (FN) and false positives (FP), test accuracy (Acc), and Matthews correlation coefficient (MCC) were calculated for the biomarkers using the best cut-off.

Biomarkers	AUC (95% Confidence Interval)	*p*-Value	Cut-Off (Youden’s Index)	Se (%)	Sp (%)	FN (%)	FP (%)	Acc (%)	MCC
aMMP-8 PoC test	1.000 (1.000–1.000)	<0.001	–	100.0	100.0	0.0	0.0	100.0	1.000
aMMP-8 (IFMA) (ng/mL)	0.829 (0.715–0.943)	<0.001	29.4	76.9	80.8	22.2	20.0	78.9	0.577
			20.0	80.8	61.5	23.8	32.3	71.2	0.431
MMP-8 per TIMP-1	0.787 (0.663–0.911)	<0.001	0.27	80.8	65.4	22.7	30.0	73.1	0.468
Act MMP-9	0.518 (0.358–0.677)	0.826	0.16	96.2	19.2	16.5	45.6	57.7	0.241
Pro MMP-9	0.598 (0.437–0.758)	0.227	0.66	92.3	42.3	15.4	38.5	67.3	0.400
PMN elastase (ng/mL)	0.765 (0.630–0.900)	0.001	20.2	73.1	76.9	25.9	24.0	75.0	0.500
MPO (ng/mL)	0.763 (0.632–0.894)	0.001	238.2	65.4	80.8	30.0	22.7	73.1	0.468
BOP (yes/no)	0.712 (0.568–0.855)	0.009	0.50	65.4	76.9	31.0	26.1	71.2	0.426
TIMP-1 (ng/mL)	0.593 (0.434–0.753)	0.249	36.8	73.1	53.8	33.3	38.7	63.5	0.274
